# Impact of ED Organization with a Holding Area and a Dedicated Team on the Adherence to International Guidelines for Patients with Acute Pulmonary Embolism: Experience of an Emergency Department Organized in Areas of Intensity of Care

**DOI:** 10.3390/medicines7100060

**Published:** 2020-09-24

**Authors:** Gabriele Savioli, Iride Francesca Ceresa, Paolo Maggioni, Massimiliano Lava, Giovanni Ricevuti, Federica Manzoni, Enrico Oddone, Maria Antonietta Bressan

**Affiliations:** 1 Emergency Department, Irccs Policlinico San Matteo, 27100 Pavia, Italy; irideceresa@gmail.com (I.F.C.); paolo.maggioni01@universitadipavia.it (P.M.); mita.bressan@gmail.com (M.A.B.); 2 PhD School in Experimental Medicine, Department of Clinical-Surgical, Diagnostic and Pediatric Sciences, University of Pavia, 27100 Pavia, Italy; 3 Neuro Radiodiagnostic, Irccs Policlinico San Matteo, 27100 Pavia, Italy; m.lava@smatteo.pv.it; 4 Department of Drug Science, University of Pavia, Italy, Saint Camillus International University of Health Sciences, 00131 Rome, Italy; giovanni.ricevuti@unipv.it; 5 Clinical Epidemiology and Biometry Unit, Irccs Policlinico San Matteo, 27100 Pavia, Italy; f.manzoni@smatteo.pv.it; 6 Department of Public Health, Experimental and Forensic Medicine, University of Pavia, 27100 Pavia, Italy; enrico.oddone@unipv.it

**Keywords:** emergency department, emergency room, pulmonary embolism, adherence to guidelines, guidelines, decision making, holding area, decision area, intensive brief observation area

## Abstract

**Background:** Adherence to guidelines by physicians of an emergency department (ED) depends on many factors: guideline and environmental factors; patient and practitioner characteristics; the social-political context. We focused on the impact of the environmental influence and of the patients’ characteristics on adherence to the guidelines. It is our intention to demonstrate how environmental factors such as ED organization more affect adherence to guidelines than the patient’s clinical presentation, even in a clinically insidious disease such as pulmonary embolism (PE). **Methods:** A single-center observational study was carried out on all patients who were seen at our Department of Emergency and Acceptance from 1 January to 31 December 2017 for PE. For the assessment of adherence to guidelines, we used the European guidelines 2014 and analyzed adherence to the correct use of clinical decision rule (CDR as Wells, Geneva, and YEARS); the correct initiation of heparin therapy; and the management of patients at high risk for short-term mortality. The primary endpoint of our study was to determine whether adherence to the guidelines as a whole depends on patients’ management in a holding area. The secondary objective was to determine whether adherence to the guidelines depended on patient characteristics such as the presence of typical symptoms or severe clinical features (massive pulmonary embolism; organ damage). **Results:** There were significant differences between patients who passed through OBI and those who did not, in terms of both administration of heparin therapy alone (*p* = 0.007) and the composite endpoints of heparin therapy initiation and observation/monitoring (*p* = 0.004), as indicated by the guidelines. For the subgroups of patients with massive PE, organ damage, and typical symptoms, there was no greater adherence to the decision making, administration of heparin therapy alone, and the endpoints of heparin therapy initiation and guideline-based observation/monitoring. **Conclusions:** Patients managed in an ED holding area were managed more in accordance with the guidelines than those who were managed only in the visiting ED rooms and directly hospitalized from there.

## 1. Introduction

### 1.1. Background

Venous thromboembolism is the third most frequent cardiovascular disease, with a reported overall annual incidence of 100 to 200 per 100,000 inhabitants, and may be lethal in the acute phase or lead to chronic disease and disability [1,2,3,4,5,6,7,8,9,10,11,12,13,14,15,16,17]. Studies have shown that venous thromboembolism remains underdiagnosed and that more inclusive diagnostic algorithms are needed. In this direction, European guidelines have underscored the relevance of developing predictive indices for patients who are clinically suspected to have pulmonary embolism (PE) [18,19]. The guidelines showed how stratification of mortality risk and rapid identification of massive PE were crucial for the correct and immediate treatment of patients. Therefore, good adherence to the guidelines is important. However, data on adherence to European guidelines in clinical practice, particularly in the acute setting of the emergency department (ED), have been unclear.

More generally, many evidence-based practice guidelines exist in health care, but adherence to these guidelines is generally low among care professionals [20,21,22]. Lack of adherence to practice guidelines can lead to omission of necessary care and contribute to preventable harm, suboptimal patient outcomes, or poor resources utilization [22]. Thus, knowing the level of adherence to practice guidelines is important for improving the quality of patient care and can also help decrease variability in treatment. Various models have been developed which demonstrate that adherence to guidelines can be influenced by multiple factors, such as (1) patient characteristics, (2) practitioner characteristics, (3) guideline and (4) environmental factors, and (5) the social-political context [23,24,25,26,27,28,29,30,31,32,33,34,35,36,37].

Patient characteristics, such as patient comorbidities, the presence of typical symptoms, severity of sickness, and awareness of need, can affect adherence to guidelines [34,35,36]. The most insidious and unusual clinical manifestations can lead to diagnostic delays, misdiagnosis, or be confusing factors for physicians.

As for practitioner characteristics, it is important that training is continuous and up-to-date. This is facilitated by courses, and active training events should be developed that are tailored to professionals’ needs to overcome the perceived barriers [26,27,28,29,30,31,32,33,34,35].

As for environmental factors, it should be emphasized how important environmental factors are for ED layout. The emergency physician is often working in an overcrowded ED. Emergency department (ED) overcrowding has become a serious and growing problem common around the world, with significant worldwide public health problems. Overcrowding is the product of several hospital internal and external factors, and the most relevant are the insufficient access to hospital beds and shortage of ED nursing and physician staff. Overcrowding can lead to poor adherence to approved guidelines [38,39,40,41,42,43,44,45,46,47,48,49,50,51]. Overcrowding can also lead to patients’ poor outcomes, adverse events, increased morbidity and mortality, and overall inferior health care [52,53,54,55,56,57,58,59,60,61,62,63,64,65,66,67,68,69,70]. Often, crowding is due to access block, which has been defined as a delay in bringing admitted patients to the inpatient beds at the wards [52,71,72,73,74]. For several decades, emergency department (ED) crowding has been extensively discussed and various interventions to reduce the ED crowding were suggested [75,76,77,78,79,80,81,82,83].

One of the answers to this problem is the creation of holding units within the ER where patients can be stabilized and managed more appropriately. Holding units are able to alleviate access blocks and ED overcrowding, reduce hospitalization, and improve care [52,84,85,86], and have been shown to play a role in selected clinical conditions, such as acute exacerbation of heart failure [87,88,89,90,91,92]. However, the role of observation units among patients with acute PE remains unclear [93]. In EDs that use a triage system based on the medical examination of patients who come in for consult, patients are categorized according to level of care needed and are prioritized to either a medium- to high- or a low-intensity path of care. Subsequently, patients are either directed to a holding/observation area, discharged, or admitted to the hospital wards.

### 1.2. Aim

As guideline characteristics and the social-political context are to be considered constant in the same hospital and referring only to one adopted guideline, we focused on the impact of the patients’ characteristics and on the environmental influence on adherence to the guidelines.

As regards the physicians’ characteristics, in our ED, there were multiple meetings and active events presenting the European Guidelines and the company’s therapeutic diagnostic pathway on pulmonary embolism derived from them. As doctors have responded in a participatory manner and successfully, we believe that this possible confusing factor is therefore attenuated or eliminated.

Research into the influence of ED organization on adherence to guidelines matches the theory of “latent errors” coined by James Reason [94,95,96]. Latent errors (or latent conditions) refer to less apparent failures of organization or design that contribute to the occurrence of errors or allow them to cause harm to workers. For instance, whereas the active failure in a particular adverse event may have been a mistake in programming a logic controller, a latent error might be that the institution uses multiple different software codes, making programming errors more likely. Thus, latent errors are quite literally “accidents waiting to happen”.

In particular, we hypothesized that adherence to the guidelines depends on ED organization with a holding area.

We believe that adherence to the guidelines on acute PE at EDs would depend on the pathway followed by the patient and the allocation, albeit transient, to a holding area. In Italian EDs, these holding areas are called Intensive Brief Observation (OBI) areas.

We also wanted to see if adherence depended on typical and serious clinical manifestations.

## 2. Materials and Methods

### 2.1. Overall Design

A single-center observational study was carried out on all patients who were seen at the Department of Emergency and Acceptance of the IRCCS Polyclinic San Matteo Foundation of Pavia from 1 January to 31 December 2017 for PE. We conducted a study whose data were collected in a restrictive manner.

Eligible patients were identified in the electronic database, based on discharge diagnosis codes that corresponded to PE. For each patient, personal and clinical data were extracted from the PIESSE digital platform, and the electronic record was examined individually to determine eligibility for inclusion in the study. The electronic folder displayed information collected and reported by the ED physician, clinical reports carried out by medical specialists, nursing diaries, and results of laboratory and radiologic examinations.

Demographic data (i.e., sex and age); risk factors for PE; duration of waiting, processes, and stay in the Hospital (LOS); blood pressure; means of arrival; entry and exit codes; hematologic examinations, such as hemoglobin, Arterial Blood Gas test (ABG), and clotting time; and rates of hospitalization and death were collected. All performance folders were viewed and evaluated, and all CTs were thoroughly reviewed by a single experienced radiologist.

### 2.2. ED Organization: Access Criteria to the Various Intensity of Care and to OBI

Our ED is divided into areas of intensity of care, including low and medium to high. Patients who arrive at our ED are first subjected to triage, where specialized nurses with basic and advanced business training courses collect information two times on each patient’s demographic data, chief complaint, and short history; thereafter, the patients are asked to proceed for determination of vital signs and visual inspection. At this stage, using the written protocols or triage grids based on the evolution of the main symptom, medical history, and vital signs, patients are assigned a priority code for medical examination and process flow to the area of the needed intensity of care.

The criteria for triage to medium-to-high intensity of care include abnormal vital signs or consciousness; the evolutionary risk of any concomitant symptoms, such as typical chest pain; and the need for noninvasive ventilation and multiparameter monitoring.

After triage, each patient is brought inside the ED room for examination by a doctor who decides on the patient’s therapeutic/diagnostic pathway. Patients in both the low and medium-high areas of intensity of care have access to a stabilization area called the OBI.

Patients are assigned to the OBI group or the non-OBI group through a clinical evaluation, which aims to include those who were in a worse clinical condition in the first group. Patients who need i.v. therapy, non-invasive ventilation, cardioactive therapy (including amine), or continuous monitoring of vital parameters are sent to the OBI. Patients with the need to complete a differential diagnosis are also sent, which is thought to take more than 6 h. This group frequently includes patients who are suspected of having an acute ischemic disease of the NSTEMI type or aortic dissection (The type of patients sent to OBI, therefore, particularly benefit from BNP or TNI dosages. This optimizes ED physicians’ decision making for PE. The dosage of TNI and BNP is crucial in the management of patients with EP because it allows one to identify, according to the guidelines, patients at high risk of mortality. We recall how patients are considered to be at high risk of short-term mortality and therefore, deserving of multiparameter monitoring and possible thrombolysis rescue when they present both right ventricular dysfunction on echocardiography or CT and positive markers of myocardial injury.). Moreover, a further criterion of inclusion considers patients clinically judged to need hospitalization in the medical department but who are not yet stable hemodynamically. Patients in need of hospitalization for which a bed in the ward is not quickly available are sent also to the OBI. Additionally, patients are excluded due to peri-arrests or cardiac arrest with ongoing resuscitation maneuvers. At the end of 2016, a team of doctors from our ED team was chosen to comprise the OBI team, which had the mission of discharging patients safely or hospitalizing those who needed it, as appropriate, as well as managing the bed allocations of all emergency admissions.

However, based on their assessment, the doctors at the ED room can decide to admit a patient directly to hospital ward without passing through the OBI.

At the end of the process, depending on disease severity and stabilization, patients are admitted, discharged, or transferred to a hospital that has a lower intensity of care.

### 2.3. Inclusion and Exclusion Criteria

This study included all patients aged >18 or <100 years and those who had acute PE with a state of consciousness not altered, ability to read, and consent to the processing of data for health and research purposes.

### 2.4. Outcomes

The primary endpoint was adherence to the guidelines as a whole, according to environmental factors: the patient flow/ED organization with OBI, if managed in high- or low-intensity care areas at the ED, and the transit to the OBI. The secondary endpoint was adherence to the guidelines, according to patients’ characteristics, such as the presence of massive PE, of organ damage, or typical symptoms.

#### 2.4.1. Primary Outcome

For the primary outcome, we analyzed each patient’s flow to the adherence to the guidelines:

1. Patients who were managed in the low-intensity care area compared to those in high-intensity care area.
2. Patients who were managed in OBI compared to those were not.

#### 2.4.2. Secondary Outcomes

For the secondary outcomes, we analyzed the following subgroups (“clinical subgroups”):

1. Those with organ damage, such as lung infarction, right ventricular dilatation, and dilation of the pulmonary artery, compared to those without organ damage.
2. Those with massive PE, which was defined as the presence of thrombi in at least two lobar lung branches of the pulmonary artery and/or the presence of thrombi at the level of the pulmonary arterial trunks or in >50% of the pulmonary arterial bed, compared to those with peripheral PE (non-massive PE).
3. Those with typical symptoms, such as chest pain and dyspnea, and the presence of concurrent signs of deep vein thrombosis DVT, compared to those with atypical symptoms.

#### 2.4.3. Assessment of Adherence to Guidelines

We used the European Guidelines in 2014 and analyzed adherence to the following:

1. Clinical decision rule (CDR): direct performance of chest computed tomography (CT) for patients at high risk for PE or D-dimer level for those at low to intermediate risk; for the latter, a CT scan was done only if the D-dimer was positive. The CDRs used were the Wells score, YEARS score, and Geneva score.
2. Initiation of heparin therapy, as recommended by the existing guidelines at the ED and based on the SPESI mortality risk. Low molecular-weight heparin was given for low or intermediate risk, whereas fractional heparin was given for high SPESI ≥ 1 and in the presence of both right ventricular dysfunction on echocardiography or CT and positive markers of myocardial injury.
3. Monitored bed observation for patients at high risk for short-term mortality, for which the guidelines suggest observation and fractional intravenous heparin and possible thrombolysis rescue. This is a composite end point of adherence to therapy and observation. At our hospital, all medium-to-high-intensity beds, such as those for resuscitation, OBI, and high-intensity of care, were considered suitable for observation. In particular, observation was recommended for patients with high short-term mortality risk (SPESI ≥ 1) and those who had both right ventricular dysfunction on echocardiography or CT and positive markers of myocardial injury.

All the collected data were stored in a spreadsheet using the Microsoft Excel program and were later on used for statistical analysis.

### 2.5. Statistical Analysis

Continuous variables were described as mean ± standard deviation, whereas qualitative variables were expressed as counts and percentages Comparisons between two groups for continuous variables were made using Student’s *t*-test or a similar nonparametric Mann–Whitney U-test. The associations between qualitative variables were analyzed using Fisher’s exact test. All tests were two-tailed, and the significance level was set at alpha −0.05, with statistical significance set at p value of <0.05. The analyses were conducted with STATA software, version 14 (Stata Corporation, College Station, 2015, TX, USA).

## 3. Results

In total, 113 identified patients were enrolled and eligible to participate in the study. The main features of the two groups are reported in Table 1. The average age of the selected patients is 68 years, from a minimum of 19 to a maximum of 98 years. A total of 46.9% of patients are male (53 patients) and 53.10% are female (60 patients).

There were no significant differences between the groups for vital signs for patients who were managed in low-intensity care compared to those in high-intensity care. The same can be said for patients who were managed in OBI compared to those were not.

There were no significant differences for vital signs also between clinical subgroups.

### 3.1. Primary Outcome

#### 3.1.1. Level of Care Needed Area

The patients who were managed in low-intensity care and those in high-intensity care had no significant differences in terms of the CDRs. Specifically, this is true for CDR Geneva (*p* = 0.76), CDR YEARS (*p* = 0.371), and CDR WELLS (*p* = 0.370) (see Table 2).

The patients who were managed in low-intensity care and those in high-intensity care had no significant differences also with regard to heparin therapy (*p* = 0.495) (see Table 3) and for the composite end point of heparin therapy initiation or observation/monitoring (*p* = 0.307) (see Table 4), according to the criteria described in Section 2.4.3.

#### 3.1.2. Patient Flow in the OBI

The patients who passed through the OBI were treated more in line with the guidelines. The patients who passed through the OBI were those who did not have significant differences in terms of initiation of heparin therapy alone (*p* = 0.007) (see Table 3) and the composite endpoints of heparin therapy initiation or observation/monitoring (*p* = 0.004) (see Table 4), according to the criteria described in Section 2.4.3.

### 3.2. Secondary Endpoints

#### 3.2.1. Presence of Massive PE

Patients with massive PE and those with peripheral PE had no significant differences in terms of the CDRs. Specifically, this is true for CDR Geneva (*p* = 0.849), CDR YEARS (*p* = 1), and CDR WELLS (*p* = 0.444) (see Table 2). The patients with massive PE and those with peripheral PE had no significant differences also with regard to heparin therapy (*p* = 0.654) (see Table 3) and for the composite end point of heparin therapy initiation or observation/monitoring (*p* = 0.506) (see Table 4), according to the criteria described in Section 2.4.3.

#### 3.2.2. Presence of Organ Damage

Patients with organ damage and those without had no significant differences in terms of the CDRs. Specifically, this is true for CDR Geneva (*p* = 0.790), CDR YEARS (*p* = 0.355), and CDR WELLS (*p* = 0.520) (see Table 2). The patients with organ damage and those without had no significant differences also with regard to heparin therapy (*p* = 0.122) (see Table 3) and for the composite end point of heparin therapy initiation or observation/monitoring (*p* = 0.101) (see Table 4), according to the criteria described in Section 2.4.3.

#### 3.2.3. Presence of Typical Symptoms

Patients with typical symptoms and those with atypical ones had no significant differences in terms of the CDRs. Specifically, this is true for CDR Geneva (*p* = 1), CDR YEARS (*p* = 0.831), and CDR WELLS (*p* = 0.286) (see Table 2). The patients with typical symptoms and those with atypical ones had no significant differences also with regard to heparin therapy (*p* = 0.319) (see Table 3) and for the composite end point of heparin therapy initiation or observation/monitoring (*p* = 0.455) (see Table 4), according to the criteria described in Section 2.4.3.

## 4. Discussion

Our study confirms the literature data of how adherence to these guidelines is generally low (60–70%) among care professionals. Similar trends have been identified by research from Dartmouth Medical School, which indicated that 30–40% of patients in regional America do not receive care consistent with current evidence, and that 20–25% of care provided is unnecessary or potentially harmful [97]. Similar data have also been experienced in other studies [20,21,22].

Our analysis shows that adherence to the guidelines depends on environmental factor and in particular, on ED organization with a holding area: here, the adherence is much greater (about 90%).

In particular, as regards environmental factors, adherence to the guidelines does not differ if the patient is managed in the medium- or low-intensity area of care. This may depend on whether doctors’ knowledge of the guidelines is homogeneous, and let us remember that a number of ED meetings were held in which the guidelines themselves were explained. This may also depend on the fact that overcrowding affects the entire ED.

Patient management in a holding area, on the other hand, in our study correlates with greater adherence to the guidelines. In our opinion, the most salient fact to highlight is that both in OBI and in the area of medium- and high-intensity care, it was possible to perform non-invasive ventilation, continuous monitoring of patients, cardioactive therapies, and invasive care. However, only in OBI is there a significant difference in adherence to the guidelines. These results match with the literature data that holding units are able to alleviate access blocks and ED overcrowding, and improved care [38,39,40,41,42,43,44,45,46,52].

Poor adherence to approved guidelines was reported to be a consequence of ED crowding in previous studies [38,39,40,41,42,43,44,45,46,47,48,49,50] and underlined in recent metanalysis [51,97]. In particular, increased time to assessment of pain and/or delays in the administration of analgesics were found to be positively associated with ED crowding [42,43,46,47]. Similarly, some studies identified a positive association between delayed time to administration and ED crowding [40,41,44,45,49]. An American study, involving the analysis of data from a voluntary registry tracking guideline adherence, found that patients with non-STEMI who boarded for long periods of time in the ED were less likely to receive guideline-recommended therapies and were at higher risk for repeat MI [39].

As inferred from the available literature [84,85,86,98,99], organization of the ED had a strong impact on the level of adherence to guidelines and on the treatment of serious and complex pathologies, such as PE. The OBI can address the negative effects of overcrowding or boarding on patient management [52,53,54,55,56,57,58,59,84,85,86,98,99] and can be used as a decision making and stabilization area that allows a team of dedicated doctors to carry out the necessary assessments and therapies, as indicated by the guidelines. This may lead to significant improvements in clinical practice and adequate patient care. Our data, therefore, show a positive impact of environmental factors on adherence to the guidelines, in this specific case, of the organization of an ED with a holding area.

In our opinion, the advantages of having a holding area such as OBI can be attributed to many reasons, that we list below.

(a) In the non-OBI area (ED visit room), it is difficult, because of the shift-over of the patients, to guarantee the correct cadence of the intervals of emergency therapy: clinicians and nurses are busy with new cases of emergencies, which can require all of their attention. In a dedicated holding area, delicate cases partially stabilized but still in need of stabilization and close monitoring may benefit from being supported by more continuous assistance and monitoring.
(b) A dedicated observation area can keep the patient under observation for up to 24–48 h, which is unthinkable in an ER visiting room. This allows more time to frame, treat, and stabilize the patient.
(c) Observation of the clinical course and response to therapy at the OBI allowed for better stratification of risk. A longer process time would enable cardiac and chest ultrasound monitoring of the patient for careful assessment of risk and stabilization.
(d) A holding area with a team of dedicated doctors sees fewer doctors alternating in shifts than ED visit rooms. A small group of doctors who alternate in an area will more easily develop a similar and harmonious way of working and allows a greater continuity of care.

Our data underline, in our opinion, proper ED organization with a holding unit, as the OBI allows the emergency physician to give the most appropriate care to the patient and to have more adherence to the guidelines. Because lack of adherence to practice guidelines can lead to omission of necessary care and contribute to preventable harm, suboptimal patient outcomes, or poor resources utilization, it is reasonable to think, indirectly, that such an organization can improve patient care and outcomes.

This study aims to highlight the importance of organizing EDs and the participation of clinicians in the organization. In fact, in everyday real life, this can be more impactful on the adequacy of care than many other factors we are more used to focusing on.

It is our belief that efforts to have more and more fine diagnoses and increasingly targeted treatments for the patient must go hand in hand with studies that highlight what are the best organizational choices. These can be, in some areas, of great and sometimes greater relevance. This is even more true in an emergency environment, where hectic times and overcrowding can put clinicians in trouble. A careful analysis of the impact of ED organization on adherence to the guidelines may result in a correction of the latent error theorized by reason.

We also wanted to see if adherence did or did not depend on some patients’ characteristics such as typical signs and symptoms and severity of the clinical picture.

We had thought that both atypical and insidious frameworks could result in a lower adherence to the guidelines in the diagnostic phase (measured by the correct timing and execution of clinical decision rule and CT scan). We also thought the same for frameworks of less severity (the absence of organ damage and presence of peripheral pulmonary embolism), assuming that the patient himself was less engaged both at the hemodynamic level and as an intensity of symptoms. On the other hand, more serious clinical frameworks may result in greater patient engagement regardless of the typicality of the symptom. For example, a patient with breathlessness has a typical symptom. A dyspneic patient with massive pulmonary embolism and right ventricular dilation we thought could have such a clinical presentation, even in the presence of the same symptom, to make clinical suspicion greater and thus, speed up the diagnostic pathway and greater adherence to the guidelines.

The same could be thought for patients with blood marker positivity. It is known that acute dyspnea is one of the most common presentations to emergency departments [100]. The ability to distinguish between respiratory and cardiac causes of dyspnea remains an ongoing diagnostic problem in this setting. B-type natriuretic peptide (BNP) and the N-terminal peptide of its precursor, pro-BNP, along with TNI and hs-TNI, have been proposed as useful markers in helping distinguish between cardiac and noncardiac causes [101,102,103,104,105,106,107,108,109,110]. An earlier accurate diagnosis might lead to earlier and more appropriate therapy and might reduce hospital admissions, shorten length of inpatient stay, and decrease mortality. B-type natriuretic peptide has been proposed as a useful surrogate primarily for left ventricular wall distention and cardiac pressures [111]. In particular, a systematic review showed that the use of BNP testing in the emergency department for patients presenting with dyspnea of suspected cardiac origin moderately reduced the length of hospital and intensive care unit stay but had no clear effect on hospital and critical care admission rates or mortality [112].

We also thought that a diagnosis of a more severe degree of pulmonary embolism could, in itself, activate the clinician to a greater adherence to the guidelines, as far as therapy is concerned. This is also because it is known that more serious clinical forms of a disease have been shown to coincide with worse outcomes, and not only in cases of pulmonary embolism, but also for other pathologies [94,95,96].

However, these were not reflected in our results.

In part, this may be due to the size of the sample of patients we enlisted. In part, it could be an effect of active training meetings that emphasized the atypical symptoms and insidiousness of the clinic. However, overseas and Australian reports indicate that variation in clinical practice is common, even where agreed clinical practice guidelines exist [113,114,115,116,117]. It has already been pointed out that wide, unwarranted variations that cannot be explained by illness severity or patient factors are frequent, and clinical practice is often idiosyncratic and unscientific [21,27].

There are multiple, diverse reasons behind variation in clinical practice, reflecting personal and organizational levels. The reasons why gaps occur between evidence and practice are complex, and efforts to improve uptake are unlikely to be successful if they are one-dimensional; focus on individual health professionals; and do not take into account the different organizational solutions [100,118]. The study of adherence to practical guidelines in real-life clinical practice must always be a major consideration if successful implementation is to occur.

### 4.1. Future Perspectives

This model that we applied to our reality can be applied in settings that do not have an ED and have limited availability of beds for medium intensity of care at the hospital. For the best outcomes and management of available health resources, we propose a model in which a dedicated team, perhaps rotating, takes care of both stabilizing complex patients and boarding with appropriate bed management.

### 4.2. Limitation

Our conclusions are limited by the observational nature of the study, including partly retrospective retrieval of information. Another limitation of our study is that we have not considered other patient characteristics like comorbidities. Another one is the limited number of patients enrolled.

## 5. Conclusions

Our data show how ED patients with acute pulmonary embolism managed in a holding unit such as the OBI with a dedicated team of doctors were managed more in accordance with the guidelines than those who were managed only in the ED visiting rooms and directly hospitalized from there.

## Figures and Tables

**Table 1:** The main demographic and clinical characteristics of the various subgroups analyzed. At the top, the analytic subgroups for the primary outcome. Below, the subgroups analyzed in the secondary outcomes.

Main Demographic and Clinical Characteristics of the Various Subgroups		Patient (pt) Number	Pt %	Age (Mean) (+/−st dv)	Male (%)	Female (%)	SBP (mmHg) (+/−st dv)	DBP (mmHg) (+/−st dv)	HR (bpm) (+/−st dv)	SAT O_2_ (%) (+/−st dv)
Primary Outcome	High-intensity care area	68	60.17	70	49	51	137	81	90	94
				(+/−14)			(+/−22)	(+/−13)	(+/−20)	(+/−6)
	Low-intensity care area	45	39.83	64	46	54	135	82	83	96
				(+/−19)			(+/−19)	(+/−11)	(+/−15)	(+/−3)
	OBI	47	42	69	51	49	140	81	91	94
				(+/−17)			(+/−21)	(+/−14)	(+/−19)	(+/−5)
	Not-OBI	66	58	67	44	56	135	82	86	95
				(+/−17)			(+/−22)	(+/-13)	(+/−21)	(+/−6)
Secondary Outcomes	With organ damage (OD)	50	44.25	67	45	55	139	85	90	94
				(+/−19)			(+/−23)	(+/−15)	(+/−20)	(+/−7)
	Without OD	63	55.75	72	47	53	136	79	87	95
				(+/−17)			(+/−20)	(+/−12)	(+/−20)	(+/−4)
	Massive PE	61	53.98	70	46	54	136	82	93	94
				(+/−16)			(+/−18)	(+/−13)	(+/−22)	(+/−6)
	Peripheral PE	52	46.02	65	48	52	139	81	82	96
				(+/−19)			(+/−25)	(+/−14)	(+/−16)	(+/−4)
	Typical symptoms	84	74.34	69	44	56	137	81	86	95
				(+/−17)			(+/−21)	(+/−13)	(+/−20)	(+/−5)
	Atypical symptoms	29	25.66	64	53	47	138	83	94	93
				(+/−18)			(+/−24)	(+/−15)	(+/−19)	(+/−8)

HR—heart rate; SBP—systolic blood pressure; DBP—diastolic blood pressure; SatO_2_—oxygen saturation; st dv—standard deviation.

**Table 2:** Adherence to clinical decision rule (CDR). At the top, the analytic subgroups for the primary outcome. Below, the subgroups analyzed in the secondary outcomes.

Adherence to Clinical Decision Rule (CDR)	Adherence to Clinical Decision Rule (CDR)	Geneva	Wells	Years
		Yes (%)	Yes (%)	Yes (%)
Primary endpoint	High-intensity care area	59.42 °	49.28 °	59.42 °
	Low-intensity care area	50 °	41.3 °	47.83 °
	OBI	n.e. *	n.e. *	n.e. *
	Not-OBI	n.e. *	n.e. *	n.e. *
Secondary endpoints	With organ damage (OD)	54°	44 °	58 °
	Without OD	59.68 °	50 °	54.84 °
	Massive PE	59.32 °	47.46 °	55.93 °
	Peripheral PE	55.77 °	48.08 °	57.69 °
	Typical symptoms	54.22 °	46.99 °	50.6 °
	Atypical symptoms	65.52 °	48.28 °	72.41 °

° *p* > 0.05; * not evaluable because it applies only at the beginning of the diagnostic process.

**Table 3:** Adherence to initiation of heparin therapy. At the top, the analytic subgroups for the primary outcome. Below, the subgroups analyzed in the secondary outcomes.

Adherence to Initiation of Heparin Therapy	Adherence to Initiation of Heparin Therapy	Yes (%)	No (%)	*p*
Primary outcome	High-intensity care area	69.57	30.43	0.495
	Low-intensity care area	63.24	36.76	
	OBI	89.36	10.64	0.007
	Not-OBI	66.66	33.34	
Secondary outcomes	With organ damage (OD)	64.00	36.00	0.122
	Without OD	67.19	32.81	
	Massive PE	68.33	31.67	0.654
	Peripheral PE	63.46	36.54	
	Typical symptoms	66.67	33.33	0.319
	Atypical symptoms	63.33	36.67	

**Table 4:** Adherence to monitored bed observation and therapy. At the top, the analytic subgroups for the primary outcome. Below, the subgroups analyzed in the secondary outcomes.

Adherence to Monitored Bed Observation and Therapy	Adherence to Monitored Bed Observation and Therapy	Yes (%)	No (%)	*p*
Primary endpoint	High-intensity care area	70	30	0.495
	Low-intensity care area	54	46	
	OBI	91	9	0.004
	Not-OBI	62	38	
Secondary endpoints	With organ damage (OD)	60	40	0.101
	Without OD	63	37	
	Massive PE	57	43	0.506
	Peripheral PE	56	44	
	Typical symptoms	61	39	0.455
	Atypical symptoms	62	38	
